# An innovative technological infrastructure for managing SARS-CoV-2 data across different cohorts in compliance with General Data Protection Regulation

**DOI:** 10.1177/20552076241248922

**Published:** 2024-05-15

**Authors:** Chiara Dellacasa, Maurizio Ortali, Elisa Rossi, Hammam Abu Attieh, Thomas Osmo, Miroslav Puskaric, Eugenia Rinaldi, Fabian Prasser, Caroline Stellmach, Salvatore Cataudella, Bhaskar Agarwal, Juan Mata Naranjo, Gabriella Scipione

**Affiliations:** 1 HPC Department, CINECA Consorzio Interuniversitario, Bologna, Italy; 2 Berlin Institute of Health (BIH), 14903 Charité – Universitätsmedizin Berlin, Berlin, Germany; 3 425287 Département Archivage et Services aux Données (DASD), Centre Informatique National de l'Enseignement Supérieur (CINES), Montpellier, France; 4 404336 High Performance Computing Center Stuttgart (HLRS), University of Stuttgart, Stuttgart, Germany

**Keywords:** SARS-CoV-2, COVID-19, GDPR, healthcare data, clinical research

## Abstract

**Background:**

The ORCHESTRA project, funded by the European Commission, aims to create a pan-European cohort built on existing and new large-scale population cohorts to help rapidly advance the knowledge related to the prevention of the SARS-CoV-2 infection and the management of COVID-19 and its long-term sequelae. The integration and analysis of the very heterogeneous health data pose the challenge of building an innovative technological infrastructure as the foundation of a dedicated framework for data management that should address the regulatory requirements such as the General Data Protection Regulation (GDPR).

**Methods:**

The three participating Supercomputing European Centres (CINECA - Italy, CINES - France and HLRS - Germany) designed and deployed a dedicated infrastructure to fulfil the functional requirements for data management to ensure sensitive biomedical data confidentiality/privacy, integrity, and security. Besides the technological issues, many methodological aspects have been considered: Berlin Institute of Health (BIH), Charité provided its expertise both for data protection, information security, and data harmonisation/standardisation.

**Results:**

The resulting infrastructure is based on a multi-layer approach that integrates several security measures to ensure data protection. A centralised Data Collection Platform has been established in the Italian National Hub while, for the use cases in which data sharing is not possible due to privacy restrictions, a distributed approach for Federated Analysis has been considered. A Data Portal is available as a centralised point of access for non-sensitive data and results, according to findability, accessibility, interoperability, and reusability (FAIR) data principles. This technological infrastructure has been used to support significative data exchange between population cohorts and to publish important scientific results related to SARS-CoV-2.

**Conclusions:**

Considering the increasing demand for data usage in accordance with the requirements of the GDPR regulations, the experience gained in the project and the infrastructure released for the ORCHESTRA project can act as a model to manage future public health threats. Other projects could benefit from the results achieved by ORCHESTRA by building upon the available standardisation of variables, design of the architecture, and process used for GDPR compliance.

## Introduction

The SARS-CoV-2 pandemic has highlighted the need for innovative and rapid health-related approaches with the aim to deliver quick results for the society and to foster a higher level of preparedness in healthcare.^1^ In this scenario, in 2020 the European Commission financed a series of research reference projects on the fight against SARS-CoV-2 using high-performance computing (HPC), such as the ORCHESTRA Horizon 2020 project (grant agreement No 101016167). The *Connecting European SARS-CoV-2 Cohorts to Increase Common and Effective Response to SARS-CoV-2 Pandemic* (ORCHESTRA) consortium, led by the University of Verona (Italy), brings together key European academic experts and research institutions in infectious diseases, data management and HPC involving 26 partners (extending to a wider network of 37 partners) from 15 countries inside and outside EU.^2^

The integration and analysis of the very heterogeneous characteristics of SARS-CoV-2 health data coming from many different sources such as electronic health records, retrospective and prospective patient studies, and related ‘-omics’ data (incl. genomics, proteomics, and transcriptomics) are key drivers for the progress from (population level) evidence-based medicine towards precision medicine.^3^

Nevertheless, one of the main challenges for healthcare is to maintain data confidentiality, integrity, and security as well as to protect the privacy of individuals in compliance with regulatory requirements such as the General Data Protection Regulation (GDPR) i.e. Regulation (EU) 2016/679 of the European Parliament on the protection of natural persons with regard to the processing of personal data.^4^

## Methods

The three participating Supercomputing European Centres (CINECA - Italy, CINES - France and HLRS - Germany) designed and deployed a dedicated infrastructure to fulfil the functional requirements for data management to ensure sensitive biomedical data confidentiality/privacy, integrity, and security. Besides the technological issues, many methodological aspects have been considered: Berlin Institute of Health (BIH), Charité provided its expertise both for data protection, information security and data harmonisation/standardisation.

### Infrastructure design

Three main layers composing the distributed infrastructure are identified:

- **National Data Providers**: hospitals, universities, research networks, etc. involved in the protocol studies and corresponding to the data owners.
- **National Hubs**: one at each HPC Centre; they provide support for data storage, transformation, sharing, and analysis at national level.
- **ORCHESTRA Data Portal**: the top-level portal, hosted at CINECA, foreseen as the centralised point of access for non-sensitive data and aggregated results.

National Hub (NH) is the core component of the infrastructure: it is intended to centralise both cohort and biobanking data at national level and to support storage, sharing and analysis on pseudonimysed data, as well as retrospective and prospective data ingested from the National Data Providers.

Non-functional requirements were considered to comply with technical, legal, and organisational constraints that can depend on country-specific and organisation-specific settings; information security, such as firewalls and DMZ (“De-Militarised Zone”) networks, access rights and encryption also were considered.

**Figure 1.**
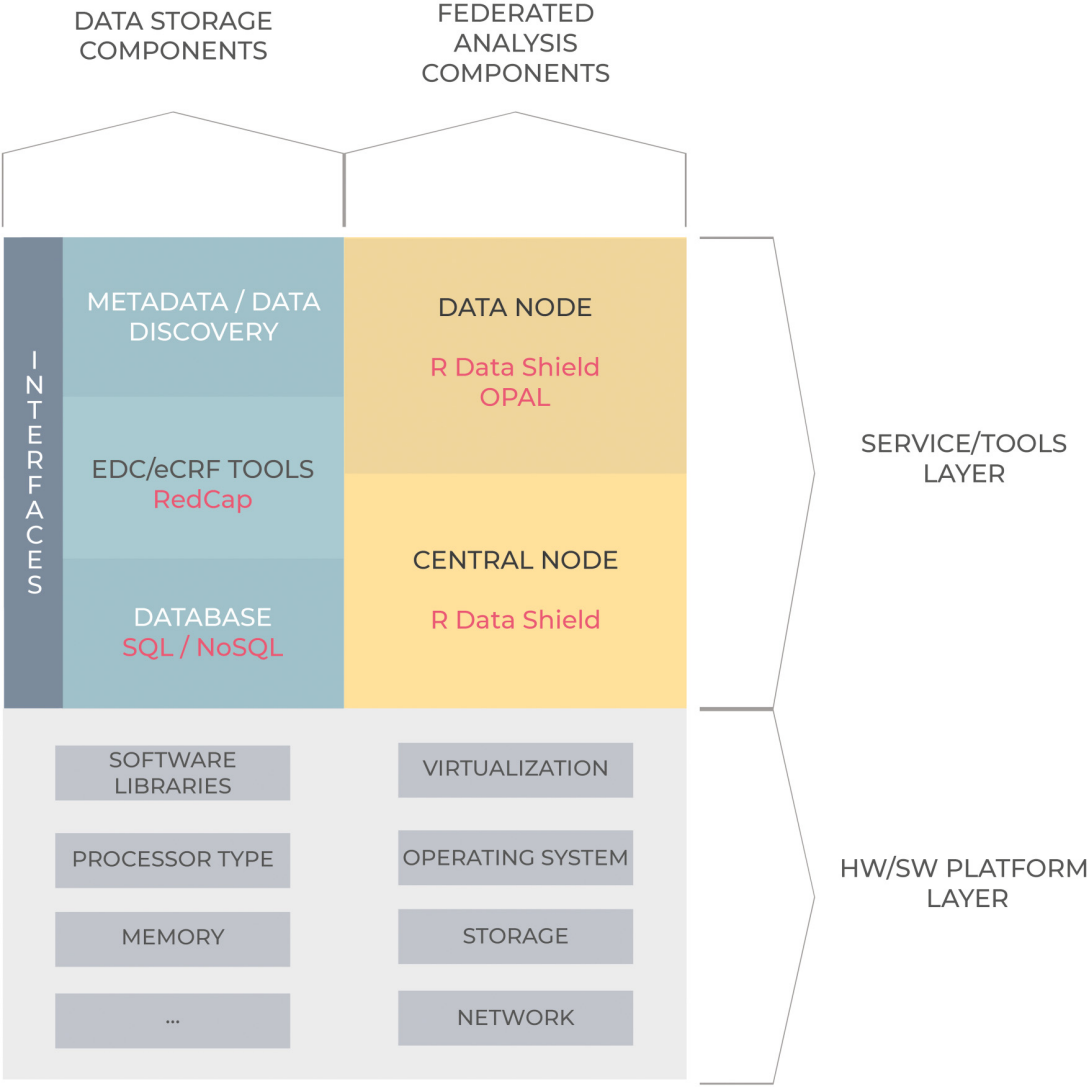
Component schema of a National Hub. It comprises two layers: a service/tools layer and an underlying hardware and operating system (HW/SW) layer.

The architecture of a NH is shown in Figure 1 and comprises two layers: a service/tools layer and an underlying hardware and operating system layer.

The service/tool layer comprises components related to data storage and tools for Federated Analysis. The infrastructure supports the findability, accessibility, interoperability, and reusability (FAIR principles) of data to facilitate data exchange and integration.^5^

Data coming from retrospective studies are directly stored in a dedicated database such as MySQL, MongoDB, or PostgreSQL. The Electronic Data Capture (EDC) Tool REDCap^6^ is adopted for data collection of prospective studies.

The service/tool layer is completed by a set of Federated Analysis open-source components: DataSHIELD,^7^ to coordinate Federated Analysis, and Opal,^8^ for data storage, whereas data providers will host Opal server which will be directly accessible from the NH's DataSHIELD.

For the hardware and software layer, each NH adopted a specific solution considering the underlying IT infrastructure, but all the three HPC centres are running Linux operating systems and make large use of virtualisation technologies, such as containerisation. A Firewall is configured to provide a high level of security by filtering all inbound and outbound traffic and only allow connections from authorised hosts, i.e. other key components such as National Data Providers and Data Portal. Disk encryption is also configured by adopting Linux Unified Key Setup (LUKS) standards whereas protection against failures of physical drives is implemented by using technologies such as RAID storage virtualisation to improve reliability.

### Data protection and information security measures

To protect the privacy of patients and study participants, the design and implementation of the NH involves data at different levels of aggregation (raw pseudonymised, anonymous, and aggregated data) coming from different countries and cohorts. A specific task dedicated to “Data Protection and Access Policy Assessment” was foreseen in the ORCHESTRA project. This task worked on defining safeguards to protect data at all stages of the data flows and processing, including guidance on when anonymisation or pseudonymisation should be used.

To define which centres were able to share data with NH and at which level of aggregation, three different types of information were analysed:

1. results of a questionnaire with a short list of information required to understand the characteristics of data of the ORCHESTRA cohorts, which was distributed to the partners (e.g. prospective or retrospective study, level of data sharing, availability of structured data, etc.);
2. the Data Management Plan (DMP) which describes the data management life cycle for the data collected, processed, and generated by the ORCHESTRA project;
3. the workflows of each study across the clinical partners to clearly and technically explain how and where the data will be shared, stored, processed, and analysed from the different cohorts to the ORCHESTRA portal through the NHs.

The results of this analysis were combined with results of a literature analysis focusing on the differences in interpretation of the GDPR in relevant countries as well as with results of discussions in meetings with to better understand the sites’ requirements.

Based on these activities, several assets have been produced to create a common understanding and toolbox (ORCHESTRA Glossary on Data Protection and Data Sharing and ORCHESTRA Recommendations on Information Security Measures, ORCHESTRA Pseudonymisation Tool (OPT)) contributing to the design of technical measures as well as organisational and legal measures and policies (e.g. DMP, consent forms, and publication policies).

#### GDPR compliance activities

For centralised data collection, the National Data Providers, which act as Data Controllers, had to appoint the NH as Data Processor to manage and store sensitive or pseudonymised data in their infrastructure. According to the National regulations and GDPR, the processing of personal data follows the principles of correctness, legality, and transparency and protection of privacy rights. Data subject categories in the process are patients and healthcare workers; types of data processed are health data, genetic data, data relating to minors, and identification numbers.

As for article 35 of the GDPR, a methodologic support for the Data Protection Impact Assessment (DPIA) has been implemented. The DPIA describes the processing and safeguards implemented and helps to manage possible risks or to assess whether remaining risks are justified.^9^

In this specific context, the methodological document reports the scope and evaluation of the risk level related to the provision of the solution for software and support services to manage and store sensitive personal data in the NH. Risk assessment reported in the document derives from an analysis based on the methodology proposed by the “European Union Agency for Cybersecurity”.^10^ The tool used for DPIA was developed by the French data protection authority “Commission Nationale de l'Informatique et des Libertés”.^11^

Additionally, where needed, a detailed internal audit with internal Data Protection Officers has been performed to assess the adoption of relevant technical and organisational measures adopted in the NH to mitigate the possible risks.

#### Pseudonymisation

When processing sensitive personal health data, various laws, regulations, and best practices recommend or mandate to store and process medical data separately from identifying information. Access to directly identifying information should be granted only to authorised individuals after successful authentication. This can be achieved by implementing pseudonymisation.^12^

In the ORCHESTRA project, while considering the heterogeneous technical and legal frameworks of the relevant sites involved, a dedicated pseudonymisation tool, called OPT, was developed. It is characterised by striking a well thought out balance between functionality and rapid deployment in diverse environments. As support for pseudonymisation was urgently needed to implement the approach to data protection agreed upon in the consortium, the approach is both pragmatic and practical given the complexity of ORCHESTRA's activities while providing a high degree of protection of personal data.

The OPT is used locally at the National Data providers to pseudonymise data about patients and samples before sharing it within the NHs.

### Harmonisation and standardisation

During the project, harmonisation and standardisation of the study variables was performed to establish semantic interoperability across cohorts and institutions.

All the available ORCHESTRA study data elements were analysed with the purpose of associating each concept to a code that represents its meaning internationally and unambiguously.

In this effort, the identification of the most appropriate and widely used international standard terminologies is a necessary and important step. SNOMED CT is currently the most comprehensive health terminology in the world,^13^ and a constantly growing ontology of preferred terms and synonyms. For this reason, SNOMED CT has been widely used for coding the variables collected in the ORCHESTRA studies. However, for measurements and observations, the standard LOINC^14^ offers a higher level of specificity and has therefore been used in ORCHESTRA to code the laboratory variables. Additionally, several terms that describe genetic /sequencing analysis were coded using the NCI Thesaurus.^15^ To identify diseases and drugs, the classifications published by WHO, respectively ICD^16^ and ATC,^17^ seemed the most appropriate and were used in ORCHESTRA.

All these standard terminologies have been carefully explored to identify the best-fitting code for each ORCHESTRA variable. When no appropriate correspondence could be found, a submission process was started with the relevant standard organisation to develop a new code that could properly represent the concept.

The mapping of variables to standard terminology codes enabled us to identify similar questions that could be associated to the same code. In some cases, for prospective studies for which protocols had not yet been finalised, it was even possible, if approved by the study group, to modify the variables to make them converge to an identical structure and terminology.

The process of standardisation and harmonisation was facilitated using the Data Dictionary. The Data Dictionary is a spreadsheet in CSV format representing the structure of the database which includes all the variables that should be used to set up the electronic capture form in REDCap. In the Data Dictionary, we associated international standard codes to the variables both by adding them to the metadata and by incorporating the code in the variable ID.

Across the ORCHESTRA studies, over 3500 SARS-CoV-2-related variables (including questions and answer value sets) were analysed to search for their representation in international standard terminologies. The process led to the identification of 964 elements that were in common between at least two studies.^18^

These common data elements (CDEs) can be further updated with elements coming from new ORCHESTRA perspective studies and constitute the common dataset to investigate scientific questions across studies. Additionally, the CDE can help to build the content matter framework for performing retrospective studies.

## Results

The resulting ORCHESTRA infrastructure implements a hierarchical aggregation architecture, where data are available from Data Providers to NHs and/or Data Portal.

While collecting the technical requirements and starting the design of the whole infrastructure, two main scenarios related to data sharing emerged:

1. Data can be shared at the level of the NH, if pseudonymised.
2. Data cannot be shared within the project unless they are anonymised or aggregated.

### Centralised data collection platform

For centralised data collection, the Italian NH, CINECA, deployed a dedicated instance of the EDC tool REDCap in its infrastructure.

REDCap is a secure, web-based software platform designed to support data capture for research studies. It has an intuitive interface for validated data capture and offers advanced audit trails capabilities for tracking data changes. Another key feature of REDCap is the possibility to seamlessly export data to common statistical packages. Procedures for data integration and interoperability with external sources are also supported.^19^

REDCap has been chosen not only because of its relevance in research involving clinical data but also considering that the project partners were to a large extent familiar with this specific EDC tool.

Furthermore, REDCap provides built-in functionalities that are mandatory for GDPR compliance. For example, data encryption is applied to the physical volume where data are stored using LUKS technology. Additionally, REDCap offers logical access control and strong authentication settings: data access is available only with personal credentials and using two-factor authentication (2FA); access credentials are disabled if not used for 90 days or after five failed login attempts while the password should be complex, at least 10 characters in length, different form the previous five and renewed every 180 days.

In addition to the controls provided by REDCap, other actions are put in place according to privacy and security constraints: log files and operations are stored in a private area, to guarantee security, for a minimum period of 6 months, while a backup system is available to avoid the loss or temporary lack of the data; the amount of personal data is minimised: stored data includes only the one needed for the specific purpose of the study while the subject is identified only through a pseudonym (given by the National Data providers using the OPT); data protection mechanisms are foreseen against threats of intrusion and the action of malicious programmes on CINECA systems and a backup system is implemented to avoid the loss or temporary lack of access to the data processed.

A Vulnerability Assessment and Penetration Testing is conducted periodically (every year) on the production environment instance, to guarantee that the required level of security is fulfilled. In addition, security measures are periodically assessed against best practice and known security threats, and new functionalities to improve security are applied. The centralised Data Platform has been used to collect valuable data regarding epidemiological variations, risk factors of SARS-CoV-2 infection and its sequelae, and vaccination efficacy in different subpopulations.

Up to now, data collected in REDCap are related to 11.500 patients (as reported in Table 1) and multi-country prospective observational studies have been performed on:

- SARS-CoV-2 out- and in-patients to define post-COVID-19 syndrome by periodically assessing clinical, virological, biochemical, and immunological aspects and physical and mental quality of life from diagnosis of SARS-CoV-2 infection up to 1-year follow-up^20^;
- the protective effect of vaccination on severe COVID-19 clinical manifestation and on the emergence of post-COVID-19 conditions^21^;
- vaccination strategies for specific high-risk populations (such as haematologic cancer patients and in solid organ transplant recipients).^22^

**Table 1.** Number of patients registered in ORCHESTRA REDCap with the indication of the data provider

Data provider	Country	No. of patients
INSERM	France	4447
University of Verona	Italy	4406
University of Bologna	Italy	1383
Treviso Hospital	Italy	264
Vicenza Hospital	Italy	261
Servicio Andaluz de Salud	Spain	250
University Medical Center Groningen	Netherlands	230
COVID HOME	Netherlands	215
University of Buenos Aires	Argentina	103

### A distributed system for Federated Analysis

When data sharing is not possible due to privacy restrictions, a distributed approach for Federated Analysis has considered. This approach allows the development of statistical analysis using data that is distributed, i.e. stored in different places and never exchanged. Thus, it is designed to respect data privacy and security, while still allowing one to carry out sophisticated modelling.^23,24^

**Figure 2.**
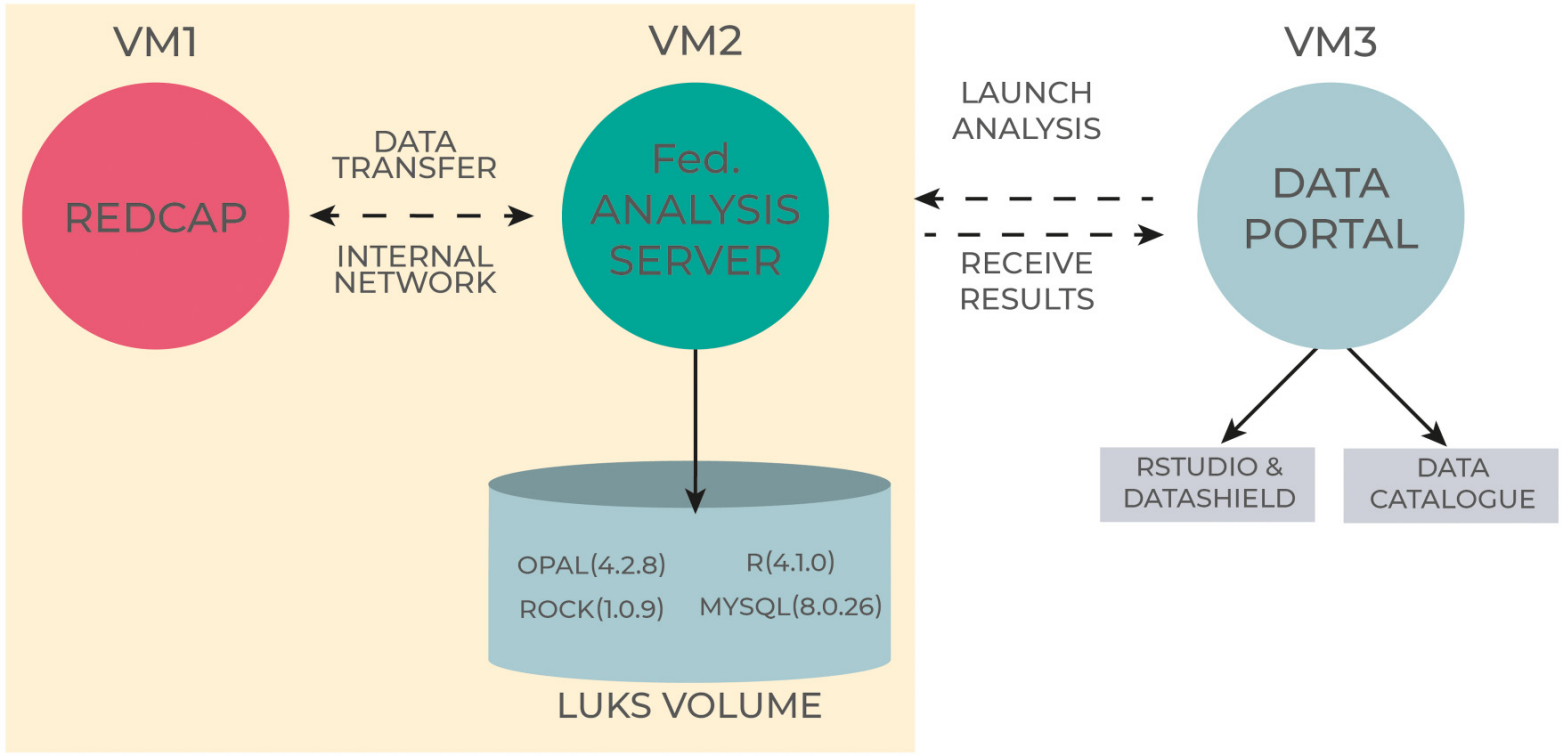
Federated Analysis architecture on CINECA resources. The first machine (VM1) is where the data is stored. The second machine (VM2) acts as the Central Node and ingests the relevant data for Federated Analysis. The third machine (VM3) is where the analysis is carried out and the results are returned.

The architecture deployed in the project is shown in Figure 2 and involves three different components (i.e. dedicated Virtual Machines - VM). These components are designed and configured to:

- manage the relevant scope of access to the sensitive data;
- minimise the security risks;
- enable an analyst to carry out Federated Analysis;
- ensure transparency between the Local Hubs, the NHs and the analysts.

The first machine (VM1) is where the data is stored. The second machine (VM2) acts as the Central Node and is linked to the VM1 to ingest the relevant data for Federated Analysis. The software stack, namely Opal, R DataShield are installed on this VM.^25^

Between VM1 and VM2, data transfer occurs through internal network to avoid any data leakage to external world. The data used to answer a specific question are ingested in Opal. Access to this machine is limited to the third VM (VM3) that we called ORCHESTRA Analyst Portal.

VM3 has RStudio and DataSHIELD installed. An analyst is given access to this machine from where a request is sent to VM2, the analysis is carried out and the results are returned, without moving data from VM2.

One example of privacy-preserving analysis includes remote analysis of the four different datasets stored in the Italian NH. The use of DataSHIELD features for merging tables based on anonymised patient ID variable was possible as a result of the data harmonisation operation done previously. Consequently, all datasets are described with standardised variable names with values assigned in a standardised format. To validate the deployed framework, Federated Analysis results have been compared with those obtained through Centralised Analysis. The overall number of patients included in the study is above 3500; summary statistics and odds ratio have been calculated. The results of the two analyses (federated vs centralised) have been compared and no differences were found.

Before the end of the project, it is expected to add more nodes (NHs or National Data Providers) and to use the Federated Analysis within the ORCHESTRA cohorts, in close collaboration with the partners involved in Data Analysis.

###  Data Portal

**Figure 3.**
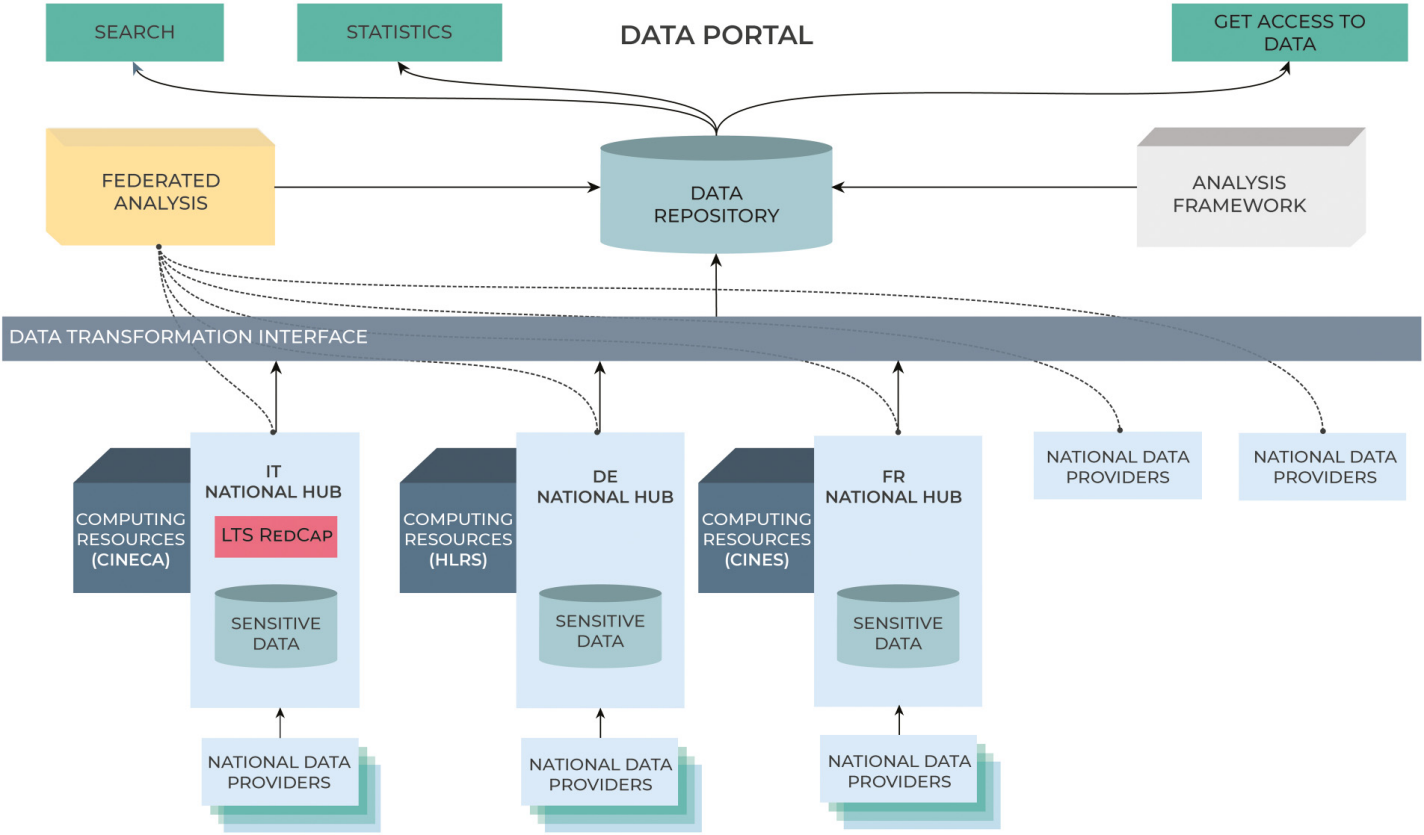
In the ORCHESTRA data management architecture, the Data Portal provides a unique point of access for non-sensitive and aggregated data and results.

The Data Portal is the top-level component in the ORCHESTRA architecture with the main role of providing a centralised point of access for non-sensitive data and results, according to FAIR data principles,^26^ as shown in Figure 3. The Data Portal aims to quickly provide all scientists, also outside the project, access to the metadata of the ORCHESTRA project, collected during the COVID-19 pandemic. Searchable datasets are as follows:

1. cohorts information provided in the DMP;
2. CDEs included in the Data Dictionary

Users are allowed to navigate and explore the data related to the cohorts and project studies: a structured database containing metadata associated with datasets and a file repository to store the corresponding file can be queried based on a set of features, and the data which satisfies the query can be downloaded.

A Statistics section allows a visual and interactive inspection of the information. For example, it is possible to dynamically build basic visualisation tools showing the results.

Authorised users can easily visualise some of the main results obtained from the Federated analysis performed in the project or they can run queries over the datasets, in respect with GDPR regulations and following the FAIR principles.

The data platform relies solely on Open-Source technologies, in particular Django and Python for the backend, Neo4j and SQLite as the main databases and HTML/JavaScript for the front-end and visual interface.

The possibility to browse ORCHESTRA metadata allows researchers to quickly evaluate if the data collected in the project would be useful for their research; data searching can be performed by type, country, population groups, and common data variables used in ORCHESTRA studies. The latter standardised, according to international standards, and homogenised, hence reducing potential bias and largely improving the data quality.

Users can request data access by filling in an online request form specifying the cohort data of interest, the variables, and the research question to pursue. After request approval, the data will be provided to the user through a Federated Analysis procedure, allowing the data owner to keep the data in their local storage place, and not physically transfer data.

Compared to other existing COVID-19-related projects such as the Covid-19 Data Portal^27^ and the SYNCHROS Cohort-Repository,^28^ the ORCHESTRA Portal does not only list the available datasets, but also hosts the cohort data that cohort owners are willing to share and provides a centralised point of access to it.

## Discussion

The SARS-CoV-2 crisis made evident the need to manage and analyse huge and heterogeneous health data coming from many different resources across different cohorts. It's a challenge that requires new computational and statistical paradigms to deal with important principles of data management and data sharing.^29^

The release of a Centralised Data Collection Platform required several activities related to the study and implementation of the procedures which are mandatory for the GDPR; national regulations can complicate the achievement of centralising health data collection while data providers should deal with their obligations to ensure data privacy and security. Pseudonymisation is a key point for health data collection; however, it is a difficult process to be implemented, both technically and in terms of procedure.^12^ In the ORCHESTRA project, considering the need to start analysing data as soon as possible, it was decided to develop a dedicated pseudonymisation tool and to provide a centralised platform for data collection on the Italian National Hub.

The use of new methodologies (such as Federated Analysis) is currently being tested and can be applied when legal constraints do not allow National Data Providers to share data within the NHs.^30^ While a federated approach is a promising avenue to maintain data privacy and ownership as data is never exchanged between the nodes, it presents its own risks and challenges.^31–33^ A well-documented risk is information leakage; other risks include model poisoning and data corruption. Extra steps at the local level must be taken to mitigate these risks. In the project, we experienced issues related to the need of trained operators and specific software and hardware capability at the level of the Data Provider. Additionally, data must adhere to a strict standardisation format to be seamlessly integrated into the model and this is a time- and cost-consuming process when data are already collected among different cohorts.^34^

The possibility to browse metadata related to cohorts and variables through the Data Portal allows researchers to quickly evaluate what is available and can be useful for their research. The Data Portal speeds up the data usage procedure while favouring the contact between the ORCHESTRA cohort owners and the applicants, thus supporting the ethical reuse of data.^35^

Nevertheless, our study experienced some limitations: GDPR constraints, ethics regulations, and the impossibility to have an agreement on data sharing in a short time. This has limited the number of cohorts included in the platform.

Furthermore, the Federated Analysis approach was applied to new-collected data, distributed in few centres. Only tabular data could be processed while it would be beneficial for research to include multiple inputs, i.e. images and tabular datasets.^30^

Data harmonisation has proven to be crucial to rapidly and easily merge data collected among different cohorts (both if the approach is centralised or distributed).^3^ In the future, one of the key factors to support across-institution research would be considering the adoption of data standardisation as a mandatory step before starting any data collection.^36^ Additionally, given the increasing risks of cybersecurity incidents and the emerging of new types of ethical risks, which are relevant along the whole data lifecycle from medical supply chain infrastructures to biomedical research, new methodologies and guidelines related to ethical concerns should be considered.^37^

Worldwide, in the case of the Covid-19 pandemic, artificial intelligence (AI) was mainly used for early disease diagnosis based on hospital records (e.g. X-ray, ultrasound images, CT, and electronic medical records)^38^; we expect that, in the event of future global pandemics, AI will be applied on data from both hospital records and clinical research with growing challenges on preserving data privacy and cybersecurity.^39^ However, AI can be efficiently applied also to defend health platforms from cyber risks, in particular by means of cyber-attack prediction and prevention of malicious use of AI itself.^40^

New frameworks will need to consider the ever-increasing use of the Internet of Things in the healthcare infrastructures^41^; the ability to move from cloud computing to real-time data analytics on the edge will solve many of the current ethical problems while preserving privacy and confidentiality.^42^

## Conclusions

The pandemic showed that the fast response of scientific research plays a significant role in fighting the disease.

Up-to-date technologies and processes such as HPC, Federated Analysis, and AI are showing their impact in the development of new innovative solutions that can be used effectively in the fields of healthcare and clinical research. The ORCHESTRA project developed an innovative data management framework to manage significative and large-scale population cohorts related to SARS-CoV-2; this framework includes guidelines, policies, and procedures for data collection, data protection and information security, data harmonisation and standardisation, and data analysis. A multi-layer infrastructure has proven to be effective to share data while addressing the need of preserving privacy and security of personal data.

The process of data harmonisation and standardisation has demonstrated to be crucial: data structured according to international standards were much easier to merge and analyse. The sharing of these standard terminologies across different projects would potentially lead to a larger base of data that can be immediately available for analysis.

The innovative solution developed in the project can be used as a model to manage data for future public health threats. It can help save time when building a new framework that should consider both the technological issues and the peculiarities related to the clinical data management; the possible integration with AI capabilities will contribute to improve the solution and support new ways of creating knowledge in healthcare.

## Supplemental Material

sj-docx-1-dhj-10.1177_20552076241248922 - Supplemental material for An innovative technological infrastructure for managing SARS-CoV-2 data across different cohorts in compliance with General Data Protection Regulation Supplemental material, sj-docx-1-dhj-10.1177_20552076241248922 for An innovative technological infrastructure for managing SARS-CoV-2 data across different cohorts in compliance with General Data Protection Regulation by Chiara Dellacasa, Maurizio Ortali, Elisa Rossi, Hammam Abu Attieh, Thomas Osmo, Miroslav Puskaric, Eugenia Rinaldi, Fabian Prasser, Caroline Stellmach, Salvatore Cataudella, Bhaskar Agarwal, Juan Mata Naranjo and Gabriella Scipione in DIGITAL HEALTH

## References

[1] Riccaboni M Verginer L . The impact of the COVID-19 pandemic on scientific research in the life sciences. PLoS ONE 2022; 17: e0263001. 10.1371/journal.pone.0263001 PMC8827464 35139089

[2] ORCHESTRA Project: https://www.orchestra-cohort.eu.

[3] Tacconelli E Gorska A Carrara E , et al. Challenges of data sharing in European COVID-19 projects: a learning opportunity for advancing pandemic preparedness and response. Lancet Reg Health Eur 2022; 21: 100467. 35942201 10.1016/j.lanepe.2022.100467 PMC9351292

[4] Assessment of the EU Member States’ rules on health data in the light of GDPR. Specific Contract No SC 2019 70 02 in the context of the Single Framework Contract. Chafea/2018/Health/03: https://ec.europa.eu/health/sites/default/files/ehealth/docs/ms_rules_health-data_en.pdf.

[5] Rinaldi E Dellacasa C Puskaric M , et al. International clinical research data ecosystem: from data standardization to federated analysis. Stud Health Technol Inform 2023; 309: 133–134. PMID: 37869823. 37869823 10.3233/SHTI230757

[6] Harris PA Taylor R Thielke R , et al. Research electronic data capture (REDCap)--a metadata-driven methodology and workflow process for providing translational research informatics support. J Biomed Inform 2009; 42: 377–381. Epub 2008 Sep 30. PMID: 18929686; PMCID: PMC2700030. 18929686 10.1016/j.jbi.2008.08.010 PMC2700030

[7] Gaye A , et al. DataSHIELD: taking the analysis to the data, not the data to the analysis. Int J Epidemiol 2014; 43: 1929–1944. Epub 2014 Sep 26. PMID: 25261970; PMCID: PMC4276062. 25261970 10.1093/ije/dyu188 PMC4276062

[8] Doiron D Marcon Y Fortier I , et al. Software application profile: opal and mica: open-source software solutions for epidemiological data management, harmonisation and dissemination. Int J Epidemiol 2017; 46: 1372–1378. PMID: 29025122; PMCID: PMC5837212. 29025122 10.1093/ije/dyx180 PMC5837212

[9] Mekovec R Peras D . Implementation of the general data protection regulation: case of higher education institution. J e-Educ e-Bus e-Manag e-Learn 2020; 10: 104–113.

[10] European Union Agency for Cybersecurity (ENISA): https://www.enisa.europa.eu/.

[11] Commission Nationale de l'Informatique et des Libertés (CNIL): https://www.cnil.fr/.

[12] Kohlmayer F Lautenschläger R Prasser F . Pseudonymization for research data collection: is the juice worth the squeeze? BMC Med Inform Decis Mak 2019; 19: 178. PMID: 31484555; PMCID: PMC6727563. 31484555 10.1186/s12911-019-0905-x PMC6727563

[13] Kate RJ . Automatic full conversion of clinical terms into SNOMED CT concepts. J Biomed Inform 2020; 111: 103585. Epub 2020 Oct 2. PMID: 33011295. 33011295 10.1016/j.jbi.2020.103585

[14] Stram M Gigliotti T Hartman D , et al. Logical observation identifiers names and codes for laboratorians. Arch Pathol Lab Med 2020; 144: 229–239. Epub 2019 Jun 20. PMID: 31219342. 31219342 10.5858/arpa.2018-0477-RA

[15] He Z Chen Y Geller J . Perceiving the usefulness of the national cancer institute metathesaurus for enriching NCIt with topological patterns. Stud Health Technol Inform 2017; 245: 863–867. PMID: 29295222; PMCID: PMC5785238. 29295222 PMC5785238

[16] Harris ST Zeng X Ford L . International classification of diseases, 10th revision: it's coming, ready or not. Health Care Manag (Frederick) 2011; 30: 227–235. Erratum in: Health Care Manag (Frederick). 2011 Oct;30(4):371. PMID: 21808174. 21808174 10.1097/HCM.0b013e318225e0a2

[17] ATC Classification: https://www.who.int/tools/atc-ddd-toolkit/atc-classification.

[18] Rinaldi E Stellmach C Rajkumar NMR , et al. Harmonization and standardization of data for a pan-European cohort on SARS-CoV-2 pandemic. NPJ Digit Med 2022; 5: 75. 35701537 10.1038/s41746-022-00620-x PMC9198067

[19] Kianersi S Luetke M Ludema C , et al. Use of research electronic data capture (REDCap) in a COVID-19 randomized controlled trial: a practical example. BMC Med Res Methodol 2021; 21: 75. PMID: 34418958; PMCID: PMC8380110. 34418958 10.1186/s12874-021-01362-2 PMC8380110

[20] Gentilotti E , et al. Clinical phenotypes and quality of life to define post-COVID-19 syndrome: a cluster analysis of the multinational, prospective ORCHESTRA cohort. EClinicalMedicine 2023; 62: 102107. PMID: 37654668; PMCID: PMC10466236. 37654668 10.1016/j.eclinm.2023.102107 PMC10466236

[21] Azzini AM , et al. How European research projects can support vaccination strategies: the case of the ORCHESTRA project for SARS-CoV-2. Vaccines (Basel) 2023; 11: 1361. PMID: 37631929; PMCID: PMC10459328. 37631929 10.3390/vaccines11081361 PMC10459328

[22] Giannella M Righi E Pascale R , et al. Evaluation of the kinetics of antibody response to COVID-19 vaccine in solid organ transplant recipients: the prospective multicenter ORCHESTRA cohort. Microorganisms 2022; 10: 1021. 35630462 10.3390/microorganisms10051021 PMC9147204

[23] Casella B Riviera W Aldinucci M , et al. MERGE: a model for multi-input biomedical federated learning. Patterns 2023; 4: 100856. DOI: 10.1016/j.patter.2023.100856. 38035188 PMC10682752

[24] Sheller MJ Edwards B Reina GA , et al. Federated learning in medicine: facilitating multi-institutional collaborations without sharing patient data. Sci Rep 2020; 10: 12598. PMID: 32724046; PMCID: PMC7387485. 32724046 10.1038/s41598-020-69250-1 PMC7387485

[25] Marcon Y Bishop T Avraam D , et al. Orchestrating privacy-protected big data analyses of data from different resources with R and DataSHIELD. PLoS Comput Biol 2021; 17: e1008880. PMID: 33784300; PMCID: PMC8034722. 10.1371/journal.pcbi.1008880 PMC8034722 33784300

[26] Wilkinson MD , et al. The FAIR guiding principles for scientific data management and stewardship. Sci Data 2016; 3: 160018. Erratum in: Sci Data. 2019 Mar 19;6(1):6. PMID: 26978244; PMCID: PMC4792175. 26978244 10.1038/sdata.2016.18 PMC4792175

[27] COVID-19 Data Portal: https://www.covid19dataportal.org/.

[28] SYNCHROS Project: https://cordis.europa.eu/project/id/825884.

[29] Bentzen HB Castro R Fears R , et al. Remove obstacles to sharing health data with researchers outside of the European union. Nat Med 2021; 27: 1329–1333. 34345050 10.1038/s41591-021-01460-0 PMC8329618

[30] Dayan I Roth HR Zhong A , et al. Federated learning for predicting clinical outcomes in patients with COVID-19. Nat Med 2021; 27: 1735–1743. 34526699 10.1038/s41591-021-01506-3 PMC9157510

[31] Li T Sahu AK Talwalkar A , et al. Federated learning: challenges, methods, and future directions. IEEE Signal Process Mag 2020; 37: 50–60.

[32] Zhang K Song X Zhang C , et al. Challenges and future directions of secure federated learning: a survey. Front Comput Sci 2022; 16: 165817. 34909232 10.1007/s11704-021-0598-z PMC8663756

[33] Huth M Arruda J Gusinow R , et al. Accessibility of covariance information creates vulnerability in federated learning frameworks. Bioinformatics 2023; 39: btad531. PMID: 37647639; PMCID: PMC10516515. 10.1093/bioinformatics/btad531 PMC10516515 37647639

[34] Abbasizanjani H Torabi F Bedston S , et al. Harmonising electronic health records for reproducible research: challenges, solutions and recommendations from a UK-wide COVID-19 research collaboration. BMC Med Inform Decis Mak 2023; 23: 8. 36647111 10.1186/s12911-022-02093-0 PMC9842203

[35] Maxwell L Shreedhar P Dauga D , et al. FAIR, ethical, and coordinated data sharing for COVID-19 response: a scoping review and cross-sectional survey of COVID-19 data sharing platforms and registries. Lancet Digit Health 2023; 5: e712–e736. PMID: 37775189; PMCID: PMC10552001. 10.1016/S2589-7500(23)00129-2 PMC10552001 37775189

[36] Doetsch JN Kajantie E Dias V , et al. Record linkage as a vital key player for the COVID-19 syndemic - the call for legal harmonization to overcome research challenges. Int J Popul Data Sci 2023; 8: 2131. PMID: 37670957; PMCID: PMC10476633. 37670957 10.23889/ijpds.v8i1.2131 PMC10476633

[37] Radanliev P De Roure D Ani U , et al. The ethics of shared COVID-19 risks: an epistemological framework for ethical health technology assessment of risk in vaccine supply chain infrastructures. Health Technol 2021; 11: 1083–1091. 10.1007/s12553-021-00565-3 PMC8180363 34123697

[38] Huang S Yang J Fong S , et al. Artificial intelligence in the diagnosis of COVID-19: challenges and perspectives. Int J Biol Sci 2021; 17(6): 1581–1587. 33907522 10.7150/ijbs.58855 PMC8071762

[39] Abdulkareem M Petersen S . The promise of AI in detection, diagnosis, and epidemiology for combating COVID-19: beyond the hype. Front Artif Intell 2021; 4: 652669. 34056579 10.3389/frai.2021.652669 PMC8160471

[40] Radanliev P De Roure D Maple C , et al. Super-forecasting the ‘technological singularity’ risks from artificial intelligence. Evolving Systems 2022; 13: 747–757. 37521026 10.1007/s12530-022-09431-7 PMC9166151

[41] Radanliev P De Roure D . Epistemological and bibliometric analysis of ethics and shared responsibility—health policy and IoT systems. Sustainability 2021; 13: 8355.

[42] Radanliev P De Roure D Maple C , et al. Forecasts on future evolution of artificial intelligence and intelligent systems. IEEE Access 2022; 10: 45280–45288.

