# Supplementary material for: An innovative technological infrastructure for managing SARS-CoV-2 data across different cohorts in compliance with General Data Protection Regulation
Source: Digit Health. 2024 May 15;10:20552076241248922. doi: 10.1177/20552076241248922 (PMC11100396; doi:10.1177/20552076241248922)
Supplement: sj-docx-1-dhj-10.1177_20552076241248922 - Supplemental material for An innovative technological infrastructure for managing SARS-CoV-2 data across different cohorts in compliance with General Data Protection Regulation [file sj-docx-1-dhj-10.1177_20552076241248922.docx]

|  | ***CINECA*** | ***CINES*** | ***HLRS*** |
| --- | --- | --- | --- |
| Service/tools layer | **REDCap**  2 virtual instances (respectively for Development and Production environment) used for the data collection of prospective studies | **REDCap** Installed on 1 virtual machine using WAMP Docker image and CentOS 7 | **REDCap**  Not installed |
|  | **Database**  Mysql v8.0.26  **Federated Learning**  1 virtual machine equipped with OPAL and R-DataSHIELD software stacks and Ubuntu LTS server v20.04.  Equipped packages:   - Opal v4.2.8 - R DataSHIELD v4.1.0 - Rock v1.0.9 | **Database**  Mysql v8.0.26  **Federated Learning**  1 virtual machine equipped with OPAL and R-DataSHIELD software  Equipped packages:   - Opal v4.2.8 - R DataSHIELD v6.6.1 - Rock v1.0.9 | **Database**  MongoDB v5.01  **Federated Learning**  1 virtual machine (VM) host hosting three VMs. Each VM encompasses one of the functional units: R server, database or user interface. R server and database are part of the internal network.  Equipped packages:   - Opal v4.2.2 - R DataSHILED v4.1.0 - Rserve v1.8-8 - Rock v1.0.7 |
| HW/OS Platform layer | The HPC cloud infrastructure is based on OpenStack Wallaby and stands out by providing   - 77 computing servers each equipped with two 8260 Intel Cascade Lake 24-cores processors at 2.4 GHz and 768 GB RAM DDR4 2933MT/s - Nodes are interconnected via Ethernet 100 Gbs network - 2 TB SSD storage - 1 PB CEPH storage raw dedicated (full NVMe/SSD).   Two virtual instances (respectively for a DEV and a PROD environment) have been provided and dedicated to the deployment of the REDCap web platform, equipped with 26 CPUs and 78GB of RAM overall. The PROD instance is also equipped with a LUKS file system to provide a low level encrypted data layer. Both virtual machines are installed with the LTS Ubuntu Server 20.04 version.  For Federated Analysis one VM is equipped with 48 CPUs (Intel Xeon Processor Cascadelake, 2.3 GHz), 360 GB of RAM and a storage space of 250 GB (Extendable). | - 3 controller nodes: 1xAMD 7302P 3GHz CPU 16C/32T, 64 GB of RAM - 3 CPU Workers: 2xAMD 7502 2,5 GHz, 32C/64T, 256 GB of RAM - 1 GPU worker: 2xAMD 7502, 512GB of RAM, 2xNVIDIA Ampere A100 - Beegfs filesystem: 92TB of NVME disks - Infiniband HDR networks - Docker will be used for deploying containerized applications, and Kubernetes will be used for the workflow and virtual machine management. | Virtualization is based on QEMU hypervisor and a libvirt API. Ubuntu Server 20.04 LTS has been chosen as an operating system on all instances. Following are some of the components built into the VM Host   - Two AMD EPYC 7261 8-Core Processors - 125 GiB of working memory - 17TB of storage in RAID5 configuration - Mellanox ConnectX-4 Lx 25GbE dual-port Network card   R Server VM is assigned with 12 vCPUs and 11.1 GiB of working memory. Opal VM and Database VM are assigned with two virtual central processing units (vCPU) and 4 GiB of working memory. |

*Table S1 - Architecture details of the three National Hubs*
